# Does Electrical Stimulation Therapy Influence Synkinesis in Facial Paralysis? A Systematic Review

**DOI:** 10.1002/oto2.70231

**Published:** 2026-04-14

**Authors:** Hänel W. Eberly, Marly Aziz, F. Jeffrey Lorenz, Heather K. Schopper, Jessyka G. Lighthall

**Affiliations:** ^1^ Division of Otolaryngology–Head and Neck Surgery Yale University School of Medicine New Haven Connecticut USA; ^2^ Penn State College of Medicine Hershey Pennsylvania USA; ^3^ Department of Otolaryngology–Head and Neck Surgery Penn State Milton S. Hershey Medical Center and Penn State College of Medicine Hershey Pennsylvania USA

**Keywords:** electrical stimulation, facial paralysis, synkinesis

## Abstract

**Objective:**

Facial paralysis (FP) has aesthetic, social, and functional consequences. While electrical stimulation (ES) shows promise for peripheral nerve injury, its role in FP remains controversial due to concerns about increasing synkinesis.

**Data Sources:**

A literature review was conducted using Medline/PubMed, Cochrane, and Web of Science databases.

**Review Methods:**

Included studies evaluated the incidence or severity of synkinesis after ES in FP patients. Extracted data included demographics, ES parameters, FP etiology, synkinesis outcomes, and treatment duration.

**Results:**

Of 601 identified articles, 6 met inclusion criteria, comprising 243 patients (92 female, reported in 5 studies). Bell's palsy was the most common FP etiology (76.5%), followed by iatrogenic facial nerve injury (20.2%). Time to treatment ranged from 1 week to 7 years post‐onset. Treatment durations varied from 2 weeks to 29 months, with inter‐impulse gaps of 30 to 3000 milliseconds, phase durations of 10 to 700 milliseconds, and amplitudes of 0.5 to 27 mA. All ES was transcutaneous. Three of 5 studies reported no differences in synkinesis scores between ES and control groups. Two studies found no differences in synkinesis incidence. Only one study showed reduced synkinesis in the ES group.

**Conclusions:**

This study represents an up‐to‐date review of synkinesis in FP patients receiving ES. Evidence suggests a benefit in overall facial function for patients without significant additional risk of synkinesis. Inconsistencies in synkinesis outcomes and measurement underscore the need for further research in this area.

The facial nerve is responsible for a multitude of functions, including facial movement, expression of emotion, and actions such as eating, speaking, smiling, and speaking. Damage to the facial nerve can lead to conditions such as facial paralysis (FP) or palsy, which can have devastating effects on function, mental health and quality of life.^1^, ^2^, ^3^ Management of facial nerve deficits is complex and treatment strategies range from surgical treatment to facial physical therapy.^4^, ^5^ While patients may recover some or all of their facial muscle strength with treatment, many patients may see limited improvement and even development of distressing sequelae. One such phenomenon that can occur following FP is postparalysis synkinesis, which is theorized to be caused by aberrant regeneration of nerve fibers leading to unintentional movement in one area of the face produced by intentional movement in another.^6^, ^7^ As patients recover, abnormal facial movements (synkinesis) and tightness (hypertonicity) start to occur around 3 to 4 months after the facial nerve insult. This may worsen up to 1 year or longer then tends to stabilize. Some studies have found the rate of developing synkinesis can be as high as 55.5% in patients with longstanding FP.^6^

Neuromuscular electrical stimulation (NMES) is a technique that provides stimulation to muscles and nerves, and has been used to improve the maintenance of muscle mass and strength and selective muscle retraining in skeletal muscle.^8^ Although facial muscles contain spindle cells, the pattern of mechanoreceptors that provide proprioceptive feedback to the central nervous system is not well understood. This makes it difficult for patients to understand and grasp the extent of their facial movement.^9^ Several studies have described the utility of electrical stimulation therapy in treating FP and related conditions.^4^, ^10^, ^11^, ^12^, ^13^ However, the role of NMES in the delayed development of synkinesis, if any, is unclear. This study aims to review the literature regarding the use of NMES in patients with FP and study its potential effect on the incidence and severity of synkinesis.

## Materials and Methods

### Search Criteria

This study was conducted according to the Preferred Reporting Items for Systematic Reviews and Meta‐Analyses (PRISMA) guidelines.^14^ Detailed search strategies were developed for the following 4 databases: Pubmed/MEDLINE, Web of Science, Scopus, and the Cochrane Library. Databases were searched from inception through June 3, 2025. The search strategy used a combination of MeSH terms and keywords for the following concepts: “Electric Stimulation Therapy” and “Synkinesis.” This search strategy was modified for all databases, replacing MeSH terms with appropriate keywords and headings where appropriate. The initial search included broader terms including electroacupuncture, chronaxie, and FP. This was to ensure that the researchers did not miss any articles encompassing the broad scope of NMES. However, it is important to note that this review did not encompass the field of electroacupuncture, but rather focused on the specific area of transcutaneous electrical stimulation. All results were entered into Rayyan (http://rayyan.qcri.org), a web and mobile application for systematic reviews and meta‐analyses.

### Selection Criteria

**Figure 1:**
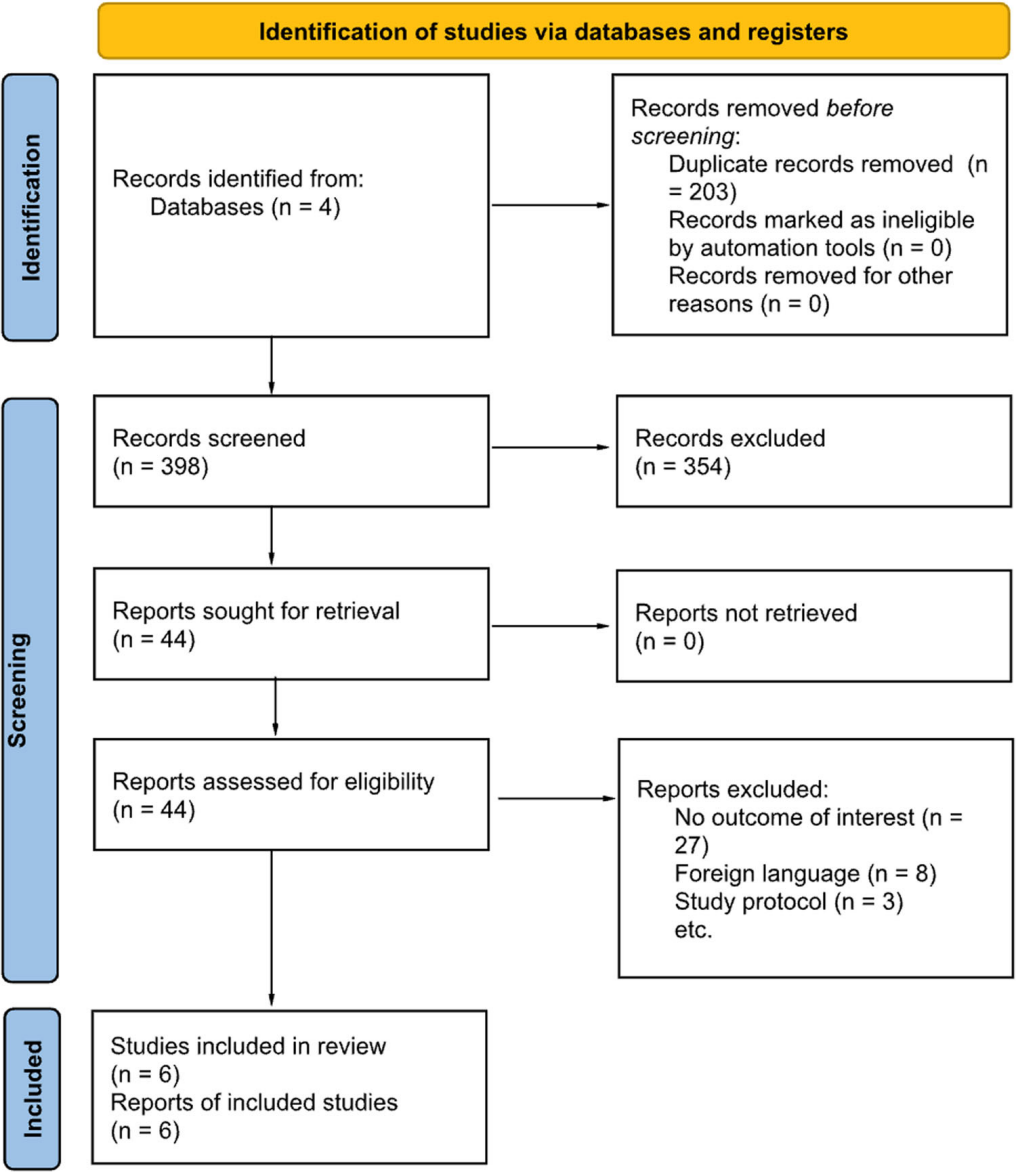
Preferred reporting items for systematic reviews and meta‐analysis (PRISMA). Flow diagram.

Studies that assessed the effect of NMES on synkinesis were included. Several articles were found that stated synkinesis was a primary outcome but included no data or discussion on synkinesis. These articles were excluded. Articles that were considered for inclusion were those in which patients underwent transcutaneous NMES therapy for FP due to any etiology (eg, Bell's palsy, traumatic injury, etc.). Articles were excluded for the following reasons: non‐English language, nonhuman studies, review articles, duplicates, inaccessible articles, and incomplete or missing statistical data. Abstracts were independently assessed by 2 reviewers (HE and MA) to identify all articles that met inclusion criteria and conflicts were resolved by a third reviewer (JL). The PRISMA diagram for inclusion and exclusion of studies can be found in Figure 1.

**Figure 2:**
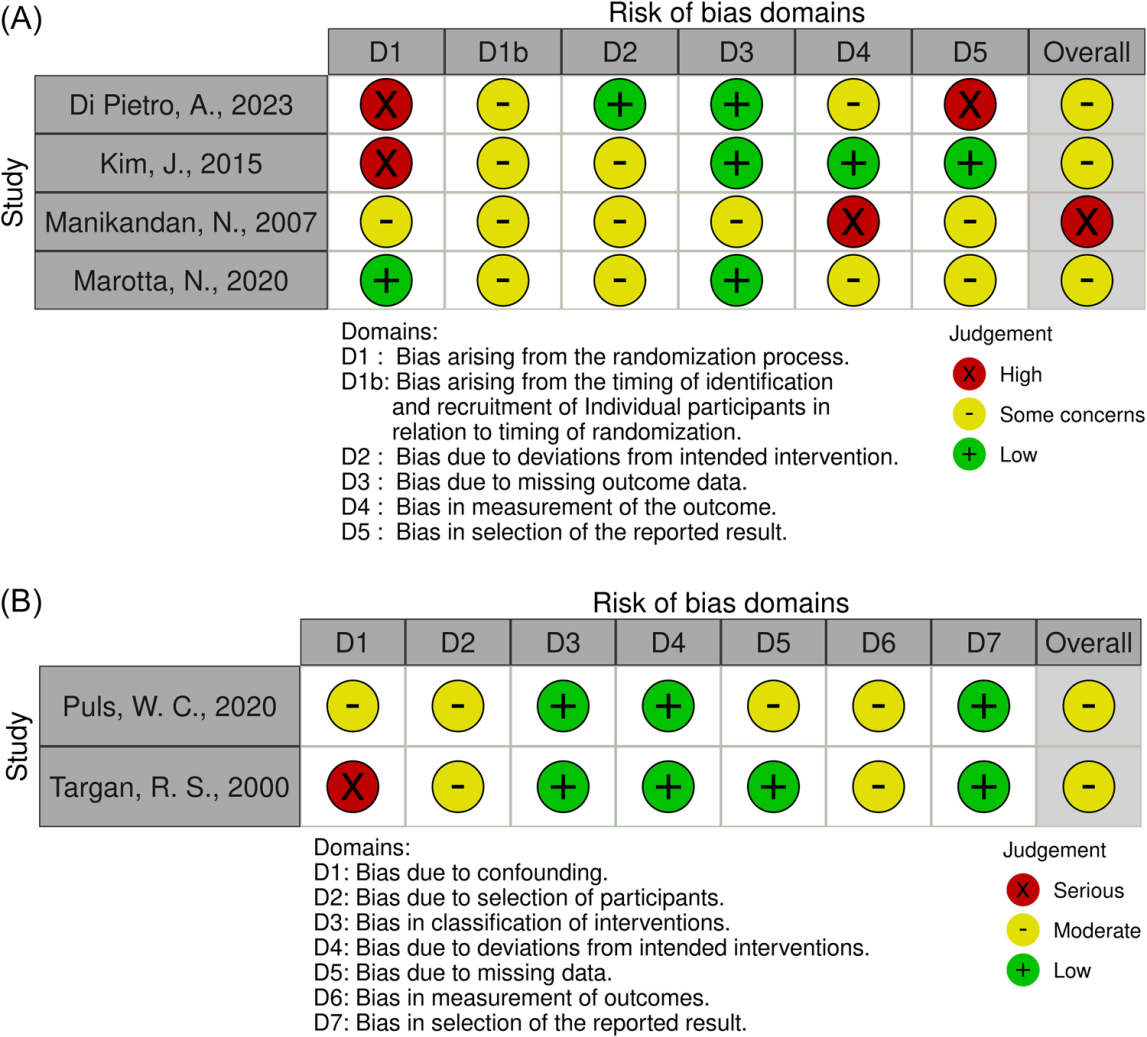
(A) Risk of Bias assessment for randomized trials using the Risk of Bias 2 (ROB2) tool. (B) Risk of Bias assessment for nonrandomized trials using the Risk of Bias in Non‐randomized Studies ‐ of Interventions (ROBINS‐I) tool.

Included articles were critically appraised to assess the level of evidence using the Oxford Center for Evidence‐Based Medicine criteria.^15^ The risk of bias was assessed according to the Cochrane Handbook for Systematic Reviews of Interventions version 6.4.^16^ The Risk of Bias in Non‐Randomized Studies–of Interventions (ROBINS‐I)^17^ tool was used to evaluate nonrandomized studies, and the Risk of Bias 2 (ROB‐2)^18^ tool was used to evaluate randomized trials. Risk of bias was assessed by 2 reviewers (HE and JL). A summary of the risk of bias assessment is found in Figure 2.

### Data Extraction

Data extracted from studies included demographics (patient age, gender); etiology of FP; time to treatment from onset of FP; treatment duration; type of NMES including inter‐impulse gap, amplitude, and phase duration; and outcomes including Sunnybrook, Facial Grading Scale, and eFACE synkinesis scores.

## Results

### Search Results and Risk of Bias Assessment

Database searches yielded 601 articles after duplicates were removed. Forty‐four articles were included after title and abstract screening, of which 6 were included in our final analysis. All studies were published between 2000 and 2024. Studies included 2 prospective cohort studies, 1 retrospective cohort study, and 3 randomized controlled trials. Of note, 1 study (Puls et al) was divided into 2 separate studies. Both were included in our analysis. Figure 1 provides a summary of the search process. The risk of bias graphs are summarized in Figure 2. Most studies were considered to have a moderate risk of bias, with 1 study having an overall high risk of bias. This was largely in part due to patient selection practices, with several articles mentioning self‐referral of patients for NMES and 1 study reporting a single‐blind study design where the participants were aware of their intervention assignment. Furthermore, the nature of grading FP is inherently subjective as most patients are graded by clinicians.

### Patient and Treatment Characteristics

There was a total of 243 patients, of which 92 were female (n = 5/6 papers). The most common etiology of FP was Bell's palsy (76.5%), followed by iatrogenic facial nerve injury (20.2%). Time to NMES treatment from onset of FP ranged from within 1 week to 7 years after onset. Two articles (n = 98 patients) investigated acute FP while 4 articles (n = 95 patients) studied chronic FP. Two articles studied incomplete FP, which has a high rate of recovery without intervention,^19^, ^20^ although no other studies specified whether the patients experienced incomplete or complete FP. Treatment duration ranged from 2 weeks to 6 months, with a range of 30 to 3000 milliseconds inter‐impulse gap, 10 to 700 milliseconds phase duration, and intensity of 0.5 to 27 mA. Five out of 6 articles used monophasic NMES pulses, with one using biphasic pulses.^10^ In many instances, intensity was titrated according to patient tolerance and varied between patients. Timeline of NMES treatment ranged from 5 to 7 days per week for 4 weeks to 6 months, with some articles listing treatment until the patient was deemed clinically improved.^19^ Descriptive features of included studies are outlined in Table 1.

**Table 1:** Descriptive Features of Included Studies

Author (Year)	Study design	Facial grading scale used	Total patients (Total females)	Mean age control	Mean/median age experimental
Di Pietro (2023)	Single‐blind controlled trial	Sunnybrook, eFACE	38 (not stated)	Not stated	36.8
Kim (2015)	Prospective randomized study	Sunnybrook, House‐Brackmann	60 (not stated)	46.5 ± 16.3	49.17 ± 18.2
Manikandan (2007)	Randomized controlled trial	Sunnybrook	56 (32)	34.61 (13.3)	35.7 (10.4)
Marotta (2020)	Randomized controlled trial	Sunnybrook	20 (6)	Not stated	42.2 ± 7.6
Puls (2020)—Study 1	Retrospective study	eFACE	45 (25)	46 (IQR 33‐61.25)	57 (IQR 45‐66)
Puls (2020)—Study 2	Retrospective study	eFACE	13 (10)	57 (IQR 42.5‐68.5)	48 (IQR 36‐66)
Targan (2000)	Prospective study	House‐Brackmann, Synkinesis score	17 (8)	Bell's: 50.4 ± 12.3	Neuroma: 45.6 ± 10.7

Abbreviation: IQR, interquartile range.

### Clinical Findings

All ES was delivered transcutaneously. Two out of 5 studies reported steroid and antiviral use prior to NMES therapy. Both studies used a regimen of prednisolone for 10 days and acyclovir for 5 days.^13^, ^20^ Sunnybrook Facial Grading Scale and eFACE synkinesis scores were used to assess symptom improvement (n = 4 papers; 64 control and 67 NMES), with 2 papers evaluating patients using the House‐Brackmann scale. Three out of 5 studies reported no differences in scores of FP grading systems between NMES and control groups.^4^, ^13^, ^19^ Two studies showed that there were no differences in the number of patients with synkinesis compared to controls prior to NMES therapy.^12^, ^20^ One study (Puls et al) demonstrated lower synkinesis scores in the NMES group for both SB scale and eFACE scales (midface and mental region).^10^ Clinical findings are outlined in Table 2.

**Table 2:** NMES Protocol and Patient Characteristics

Author (Year)	Incomplete/complete facial paralysis	Timeline from onset to treatment	Etiology of paralysis	NMES parameters	NMES protocol	Adjunctive therapy	Results
Di Pietro (2023)	Incomplete	Acute (up to 1 month after onset)	Bell's Palsy	Monophasic, pulse width 30‐200 milliseconds, frequency 5‐33.3 Hz	5 days per week for 2‐6 weeks	Neuromuscular reeducation, massage therapy	No difference in synkinesis eFACE scores between groups
Kim (2015)	Incomplete	Acute (within 1 week of onset)	Bell's Palsy	Monophasic, pulse width 50 milliseconds, frequency 20 Hz, intensity 1.4 mA average	Not stated	Prednisolone 10 days + acyclovir 5 days	One patient with synkinesis in experimental group compared to three in control group
Manikandan (2007)	Not stated	Not stated	Bell's Palsy	Not stated	6 days per week for 6 weeks	Neuromuscular reeducation, massage therapy	No improvement in Facial Grading Scale synkinesis scores. No difference in Sunnybrook synkinesis scores between groups
Marotta (2020)	Not stated	Chronic (for at least 5 months after onset)	Bell's Palsy	Monophasic and biphasic, pulse duration 700 milliseconds, frequency 80 Hz, intensity starting at 0.5 mA and increased until muscle contraction seen	5 days per week for 4 weeks	Prednisolone 10 days + acyclovir 5 days initially, short‐wave diathermy	No differences in Sunnybrook synkinesis scores between groups
Puls (2020) – Study 1	Not stated	Chronic (13 months‐7 years after onset)	Iatrogenic, other causes	Biphasic, pulse width 1000‐3000 milliseconds, pulse duration 100‐300 milliseconds, intensity 5‐27 mA	5 days per week	Hypoglossal‐facial‐jump‐anastomosis	N/a
Puls (2020) – Study 2	Not stated	Chronic (1 year after reinnervation)	Iatrogenic	Biphasic, pulse duration 100‐280 milliseconds, intensity 5‐27 mA	5 days per week	N/a	Lower synkinesis scores in NMES group for SB scale. Lower rate of synkinesis in NMES group in midface and mental region for eFACE scale
Targan (2000)	Not stated	Chronic (at least 12 months since onset)	Bell's Palsy, Iatrogenic	Monophasic, pulse width 700 milliseconds, pulse duration 86 micros, intensity submotor	Daily for 6 months	N/a	No change in number of patients with synkinesis before and after NMES

## Discussion

The incidence of synkinesis following FP varies but may as high as 55.5% in patients with longstanding FP.^6^, ^21^ The mechanism of synkinesis is thought to involve nonspecific aberrant regeneration of the nerve fibers due to ineffective myelination and neural network reorganization, leading to miscommunication between the musculature and nerves of the face.^21^, ^22^ Postparalysis synkinesis starts to develop around 3 to 4 months after the initial nerve injury, with some cases developing following facial reanimation procedures, and does not resolve.^6^, ^23^ The current treatment of FP is multifactorial and aims to minimize sequelae and maximize normal function of the face, making early and consistent treatment important to prevent sequelae such as synkinesis.^24^

NMES is considered a safe technique within the realm of electrical stimulation techniques, and has been used in clinical and scientific populations since the 19th century.^25^, ^26^, ^27^ Common side effects include pain, discomfort, skin irritation, and in severe cases burns to the skin.^25^ While past articles have recommended stopping NMES once synkinesis or contracture has appeared,^28^, ^29^ no studies have directly linked the development of synkinesis to NMES therapy. One study that surveyed 193 physical therapists in Oregon reported that common reasons for avoiding NMES included risks outweighing potential benefits, lack of training and equipment, and research showing it to be ineffective.^30^ Other general concerns cited against NMES included concern for development of abnormal movement patterns based on animal studies and personal experience as reported by several other authors.^31^, ^32^

In the present review of 6 studies (243 patients) in the literature using NMES as a treatment for FP, only 1 study demonstrated lower synkinesis scores in the NMES group compared to controls with most studies reporting no differences between groups.^10^ However, given that the efficacy of treatments for FP relies on its mechanism, acuity, and severity, it is important to evaluate the time course and presentation of each patient. For example, chronic FP due to Bell's palsy will present with a vastly different prognosis compared to iatrogenic facial nerve injury, benign tumors, neuroborreliosis, Ramsay Hunt Syndrome or even acute Bell's palsy.^7^, ^33^ Additionally, incomplete (partial) facial weakness has a high rate of recovery to normal function without intervention and without development of adverse sequelae such as synkinesis. Standard of care for acute incomplete Bell's palsy is usually corticosteroids with optional antivirals^33^ and patients are unlikely to develop synkinesis as there is no degeneration of the nerve.^7^ The utility of adding NMES in these patients is unclear, and these studies did not report statistically significant differences in eFACE synkinesis scores or number of patients experiencing synkinesis.^19^, ^20^

To assess the effect of NMES on synkinesis, it may be beneficial to study patients with acute, complete FP such as Bell's palsy, as this group is at higher risk for the development of postparalysis synkinesis.^34^ Another group that may derive the most benefit would be chronic unrecovered FP without synkinesis, refractory to other treatments. Most of the studies included in our review evaluated chronic FP but it is unclear if there was persistent weakness, or lack of movement due to chronic hypertonicity of antagonistic facial muscles, with 2 articles studying acute incomplete Bell's palsy.

The mechanism underlying electrostimulation‐mediated reinnervation is poorly understood. Possible theories include the role of activity‐dependent genetic expression in strengthening newly formed synapses, or the concentration of neurotrophic factors secreted through the stimulation of muscles in Schwann cells leading to preferential motor reinnervation.^10^, ^35^, ^36^ The possibility of axonal resprouting diminishes around 18‐24 months after onset of the injury, and absence of neural stimulation can induce atrophy and disability of musculature.^37^, ^38^ Most included articles studied patients with chronic FP^10^, ^12^, ^13^ with 2 studies studying patients with acute FP within 1 week to 1 month of onset.^6^, ^23^ However, the only study showing a decrease in synkinesis score between groups studied chronic unresolved FP (13 months‐7 years after onset).^10^ The included articles utilized low‐intensity (0.5‐27 mA) stimulation, which has been shown to induce more central nervous system input compared to higher intensities while maintaining patient comfort.^39^ Given the smaller size and potential sensitivity of facial muscles, this may be why the authors decided on lower intensity stimulation compared to high intensity stimulation.^39^

The use of NMES for FP is controversial, with some studies advocating for its use and others citing concerns of reinforcement of abnormal neural regeneration.^29^, ^30^, ^40^ A systematic review by Burelo‐Peregrino et al examining 244 patients found that patients with Bell's palsy treated with NMES demonstrated a positive response to treatment, although data on synkinesis is unavailable and the timeframe of treatment varies widely from 2 weeks to 24 months.^41^ Several other trials comparing NMES to controls demonstrate weak benefits, although again with varying timeframes ranging from weeks to years.^4^, ^20^, ^30^, ^42^ One trial did report a trend towards increased synkinesis in the NMES group over 3 months, however this was not statistically significant.^4^ One study examining frontal branch stimulation in baseline synkinetic patients demonstrated increases in eyebrow elevation to the same level as the healthy eyebrow.^43^ The study did not report any adverse outcomes regarding new‐onset or worsening of synkinesis.

Three out of 6 papers in the present review demonstrated no significant differences between control or NMES groups regarding synkinesis scores. The timeline of FP for each of these articles differed, with 1 examining chronic Bell's palsy, another acute incomplete Bell's palsy, and another that did not report timeline of FP. However, these papers were unique in their consideration of the synkinesis score as a variable. Synkinesis scores were measured using various scales including the Sunnybrook synkinesis score, which grades synkinesis on a scale from 0 to 3 on 5 movement domains measured by a trained clinician.^44^, ^45^ Other studies have used the eFACE score, which averages synkinesis subscores across various areas of the face based on clinician‐graded observations.^46^ The available literature suggests that in some cases, NMES may not affect synkinesis in certain patients, although it is important to note that the timeframe of treatment and severity or completeness of FP treated varies widely in the literature.

Other considerations regarding the use of NMES in FP is the availability, cost, and familiarity of therapists with the technique. In a study of 193 physical therapists and physical therapist assistants in the state of Oregon, therapists who do not use NMES cited lack of training, inconvenience, and a belief that NMES is ineffective as reasons it is not utilized in their practice.^30^ One often cited consideration is the worry that NMES may contribute to or worsen synkinesis.^19^, ^30^ Furthermore, of the articles screened, many described different approaches and protocols for NMES. No standard protocol currently exists, making the decision to treat with NMES largely dependent on clinician preference or untrained therapists treating FP similar to other peripheral nerve injuries. Future understanding of the impact of NMES on synkinesis would benefit from large, multicenter randomized controlled trials using a standardized set of parameters and longitudinal follow up for at least a year given the natural history of synkinesis.

### Limitations

There is an overall lack of high‐quality randomized controlled trials in the literature investigating the efficacy of NMES specifically on synkinesis in the literature. Many of the screened articles evaluated NMES but had no mention of synkinesis or related outcomes. Several case reports and foreign‐language articles were also screened out, which limits the available evidence to draw generalizable conclusions in our study. Many of the included studies also had moderate risks of bias, largely from patient selection methods which may limit our results. Data on patient comorbidities such as diabetes, tobacco use, medication use, and other coexisting medical problems was not available for analysis.

The measurement of FP and sequelae such as synkinesis are inherently subjective, as they are generally clinician graded. Although several more recent studies included the use of movement‐tracking software to provide a more objective grading, clinician‐graded scales are still the mainstay of FP measurement.^13^ The scale used across groups varies by clinician preference, making it difficult to pool results from many studies. Furthermore, the etiology and mechanism of FP differs among patients which may affect the outcome of NMES. The timeline of treatment also varied widely between studies. Patients with synkinesis tend to improve for about 4 months following onset of FP, after which synkinesis may develop and worsen over 4‐8 months post‐paralysis.^6^, ^23^ With the current results, it is difficult to comment on whether NMES has an effect on long‐term recovery and stabilization of FP following the development of synkinesis after 4‐8 months. Most screened articles focused on overall facial recovery rather than on synkinesis as a specific sequela. Additional studies may consider using validated synkinesis‐specific scales such as the synkinesis assessment questionnaire.^47^ Future research on this topic is crucial to understanding the impact of NMES on synkinesis specifically.

## Conclusion

NMES is an adjunctive treatment being used for patients with FP, with evidence suggesting a benefit in overall facial function for patients without significant additional risk of synkinesis (e.g. partial facial weakness). Based on the available literature, there is still inadequate data looking at NMES on complete FP or chronic, nonsynkinetic FP. Additionally, the timeline, severity, and etiology of FP and NMES treatment parameters vary widely in the literature with no agreed‐upon guidelines. Further research could aim to standardize NMES treatment protocols as well as studying its use in a stratified patient group.

## Author Contributions

**Hanel Eberly**, conceptualization, data collection, data analysis, writing and editing of manuscript; **Marly Aziz**, data collection; **Jeff Lorenz**, data collection, data analysis, critical editing of the manuscript; **Heather Schopper**, conceptualization, supervision, critical editing of manuscript; **Jessyka Lighthall**, conceptualization, supervision, critical editing of manuscript.

## Disclosures

### Competing interests

None.

### Funding source

None.

## References

[1] Moroco AE , Daher GS , O'Connell Ferster AP , Lighthall JG . Prevalence of body dysmorphic disorder in an otolaryngology‐head and neck surgery clinic. Ann Otol, Rhinol, Laryngol. 2023;132(7):783‐789. 10.1177/00034894221118772 35962596

[2] Saadi R , Shokri T , Schaefer E , Hollenbeak C , Lighthall JG . Depression rates after facial paralysis. Ann Plast Surg. 2019;83(2):190‐194. 10.1097/SAP.0000000000001908 31232802

[3] Vargo M , Ding P , Sacco M , et al. The psychological and psychosocial effects of facial paralysis: a review. J Plast Reconstruct Aesthet Surg. 2023;83:423‐430. 10.1016/j.bjps.2023.05.027 37311285

[4] Manikandan N . Effect of facial neuromuscular re‐education on facial symmetry in patients with Bell's palsy: a randomized controlled trial. Clin Rehabil. 2007;21(4):338‐343. 10.1177/0269215507070790 17613574

[5] Angeli SI , Chiossone E . Surgical treatment of the facial nerve in facial paralysis. Otolaryngol Clin North Am. 1997;30(5):683‐700. 9295248

[6] Salles AG , da Costa EF , Ferreira MC , do Nascimento Remigio AF , Moraes LB , Gemperli R . Epidemiologic overview of synkinesis in 353 patients with longstanding facial paralysis under treatment with botulinum toxin for 11 years. Plast Reconstr Surg. 2015;136(6):1289‐1298. 10.1097/PRS.0000000000001802 26595022

[7] Peitersen E . The natural history of Bell's palsy. Am J Otol. 1982;4(2):107‐111. 7148998

[8] Lake DA . Neuromuscular electrical stimulation: an overview and its application in the treatment of sports injuries. Sports Med. 1992;13(5):320‐336. 10.2165/00007256-199213050-00003 1565927

[9] Brach JS , VanSwearingen JM , Lenert J , Johnson PC . Facial neuromuscular retraining for oral synkinesis. Plast Reconstr Surg. 1997;99(7):1922‐1931. discussion 1932‐1933 10.1097/00006534-199706000-00017 9180715

[10] Puls WC , Jarvis JC , Ruck A , Lehmann T , Guntinas‐Lichius O , Volk GF . Surface electrical stimulation for facial paralysis is not harmful. Muscle Nerve. 2020;61(3):347‐353. 10.1002/mus.26784 31875972

[11] Wang X , Jiang F , Zhou T , Gu Q , Li X . Effectiveness of low‐frequency pulse electrical stimulation combined with dexamethasone in treating facial nerve paralysis and its impact on facial nerve function and electromyography. Altern Ther Health Med. 2024;30(12):530‐537. 38581322

[12] Targan RS , Alon G , Kay SL . Effect of long‐term electrical stimulation on motor recovery and improvement of clinical residuals in patients with unresolved facial nerve palsy. Otolaryngol Head Neck Surg. 2000;122(2):246‐252. 10.1016/S0194-5998(00)70248-8 10652399

[13] Marotta N , Demeco A , Inzitari MT , Caruso MG , Ammendolia A . Neuromuscular electrical stimulation and shortwave diathermy in unrecovered Bell palsy: a randomized controlled study. Medicine. 2020;99(8):e19152. 10.1097/MD.0000000000019152 32080092 PMC7034718

[14] Page MJ , McKenzie JE , Bossuyt PM , et al. The PRISMA 2020 statement: an updated guideline for reporting systematic reviews. BMJ. 2021;372:n71. 10.1136/bmj.n71 33782057 PMC8005924

[15] OCEBM Levels of Evidence Working Group. The Oxford Levels of Evidence 2. https://www.cebm.ox.ac.uk/resources/levels-of-evidence/ocebm-levels-of-evidence

[16] Higgins J , Thomas J , Chandler J , et al. Cochrane Handbook for Systematic Reviews of Interventions version 6.4 (updated August 2023). www.training.cochrane.org/handbook

[17] Sterne JA , Hernán MA , Reeves BC , et al. ROBINS‐I: a tool for assessing risk of bias in non‐randomised studies of interventions. BMJ. 2016;355:i4919. 10.1136/bmj.i4919 27733354 PMC5062054

[18] Sterne JAC , Savović J , Page MJ , et al. RoB 2: a revised tool for assessing risk of bias in randomised trials. BMJ. 2019;366:l4898. 10.1136/bmj.l4898 31462531

[19] Di Pietro A , Cameron M , Campana V , et al. Efficacy of adding selective electrical muscle stimulation to usual physical therapy for Bell's palsy: immediate and six‐month outcomes. Eur J Transl Myol. 2023;33(4):11630. 10.4081/ejtm.2023.11630 37877154 PMC10811644

[20] Kim J , Choi JY . The effect of subthreshold continuous electrical stimulation on the facial function of patients with Bell's palsy. Acta Otolaryngol. 2016;136(1):100‐105. 10.3109/00016489.2015.1083121 26399994

[21] Shokri T , Patel S , Ziai K , Harounian J , Lighthall JG . Facial synkinesis: a distressing sequela of facial palsy. Ear Nose Throat J. 2024;103(6):NP382‐NP391. 10.1177/01455613211054627 34836457

[22] Placheta E , Tzou CJ , Hold A , Pona I , Frey M . Facial synkinesia before and after surgical reanimation of the paralyzed face. Plast Reconstr Surg. 2014;133(6):842. 10.1097/PRS.0000000000000218 24867744

[23] Çelik M , Forta H , Vural Ç . The development of synkinesis after facial nerve paralysis. Eur Neurol. 2000;43(3):147‐151. 10.1159/000008154 10765054

[24] Owusu JA , Stewart CM , Boahene K . Facial nerve paralysis. Med Clin North Am. 2018;102(6):1135‐1143. 10.1016/j.mcna.2018.06.011 30342614

[25] Efthimiou TN , Hanel PHP , Korb S . Volunteers’ concerns about facial neuromuscular electrical stimulation. BMC Psychol. 2022;10(1):117. 10.1186/s40359-022-00827-3 35526073 PMC9080168

[26] Efthimiou TN , Perusquía‐Hernández M , Elsenaar A , Mehu M , Korb S . Application of facial neuromuscular electrical stimulation (fNMES) in psychophysiological research: practical recommendations based on a systematic review of the literature. Behav Res Methods. 2023;56(4):2941‐2976. 10.3758/s13428-023-02262-7 37864116 PMC11133044

[27] Duchenne G. *Mécanisme de La Physionomie Humaine: Où, Analyse Électro‐Physiologique de l'expression Des Passions. J.‐B. Baillière*; 1862.

[28] Mosforth J , Taverner D . Physiotherapy for Bell's palsy. BMJ. 1958;2(5097):675‐677. 10.1136/bmj.2.5097.675 13572865 PMC2026410

[29] Shafshak TS . The treatment of facial palsy from the point of view of physical and rehabilitation medicine. Eura Medicophys. 2006;42(1):41‐47. 16565685

[30] Munn A , Cameron M , Loyo M . Trends in electric stimulation for facial paralysis: electronic survey of physical therapists in Oregon. Arch Physiother Rehabil. 2020;3:001‐008.

[31] Cohen SM , Rosett BE , Shifrin DA . An analysis of independent variables affecting surgical outcomes in patients undergoing repair of maxillofacial trauma: an American College of Surgeons National Surgical Quality Improvement Program Study. J Craniofac Surg. 2017;28(3):596‐599. 10.1097/SCS.0000000000003545 28468133

[32] Skouras E , Merkel D , Grosheva M , et al. Manual stimulation, but not acute electrical stimulation prior to reconstructive surgery, improves functional recovery after facial nerve injury in rats. Restor Neurol Neurosci. 2009;27(3):237‐251. 10.3233/RNN-2009-0474 19531878

[33] Engström M , Berg T , Stjernquist‐Desatnik A , et al. Prednisolone and valaciclovir in Bell's palsy: a randomised, double‐blind, placebo‐controlled, multicentre trial. Lancet Neurol. 2008;7(11):993‐1000. 10.1016/s1474-4422(08)70221-7 18849193

[34] Rail B , Bhatia SS , Dragun AJ , et al. The natural progression of synkinesis. Plast Reconstr Surg. 2026;157:86. 10.1097/prs.0000000000012243 40492635

[35] Zealear DL , Mainthia R , Li Y , et al. Stimulation of denervated muscle promotes selective reinnervation, prevents synkinesis, and restores function. Laryngoscope. 2014;124(5):E180‐E187. 10.1002/lary.24454 24254367

[36] Brushart TM , Gerber J , Kessens P , Chen YG , Royall RM . Contributions of pathway and neuron to preferential motor reinnervation. J Neurosci. 1998;18(21):8674‐8681. 10.1523/JNEUROSCI.18-21-08674.1998 9786974 PMC6793544

[37] Li T , Wang S , Yin X , et al. Electroacupuncture with intermittent wave stimulation as rehabilitation approach for chronic Bell's palsy: a randomized controlled trial. Postgrad Med J. 2024;100(1181):151‐158. 10.1093/postmj/qgad126 38134327

[38] Biglioli F . Facial reanimations: part II‐‐long‐standing paralyses. Br J Oral Maxillofac Surg. 2015;53(10):907‐912. 10.1016/j.bjoms.2015.07.001 26194145

[39] Doucet BM , Lam A , Griffin L . Neuromuscular electrical stimulation for skeletal muscle function. Yale J Biol Med. 2012;85(2):201‐215. 22737049 PMC3375668

[40] Diels HJ . Facial paralysis: is there a role for a therapist? Facial Plast Surg. 2000;16(4):361‐364. 10.1055/s-2000-15546 11460303

[41] Burelo‐Peregrino EG , Salas‐Magaña M , Arias‐Vázquez PI , et al. Efficacy of electrotherapy in Bell's palsy treatment: a systematic review. J Back Musculoskeletal Rehabil. 2020;33(5):865‐874. 10.3233/BMR-171031 32144972

[42] Tuncay F , Borman P , Taşer B , Ünlü İ , Samim E . Role of electrical stimulation added to conventional therapy in patients with idiopathic facial (Bell) palsy. Am J Phys Med Rehabil. 2015;94(3):222‐228. 10.1097/PHM.0000000000000171 25171666

[43] Xiao R , McGonagle ER , Hadlock TA , Heaton JT . Transcutaneous facial nerve frontal branch stimulation to restore dynamic elevation of the paralyzed eyebrow in synkinetic patients. Facial Plast Surg Aesthet Med. Published online Apirl 28, 2025. 10.1089/fpsam.2025.0015 40293354

[44] Neely JG , Cherian NG , Dickerson CB , Nedzelski JM . Sunnybrook facial grading system: reliability and criteria for grading. Laryngoscope. 2010;120(5):1038‐1045. 10.1002/lary.20868 20422701

[45] Ross BG , Fradet G , Nedzelski JM . Development of a sensitive clinical facial grading system. Otolaryngol Head Neck Surg. 1996;114(3):380‐386. 10.1016/S0194-59989670206-1 8649870

[46] Banks CA , Bhama PK , Park J , Hadlock CR , Hadlock TA . Clinician‐graded electronic facial paralysis assessment: the eFACE. Plast Reconstr Surg. 2015;136(2):223e‐230e. 10.1097/PRS.0000000000001447 26218397

[47] Mehta RP , WernickRobinson M , Hadlock TA . Validation of the synkinesis assessment questionnaire. Laryngoscope. 2007;117(5):923‐926. 10.1097/MLG.0b013e3180412460 17473697

